# Investigating the Efficacy of Kidney-Protective *Lactobacillus* Mixture-Containing Pet Treats in Feline Chronic Kidney Disease and Its Possible Mechanism

**DOI:** 10.3390/ani14040630

**Published:** 2024-02-16

**Authors:** Ching-Wen Tsai, Hsiao-Wen Huang, Ya-Jane Lee, Ming-Ju Chen

**Affiliations:** 1 Department of Animal Science and Technology, National Taiwan University, Taipei 106037, Taiwan; r10626003@ntu.edu.tw (C.-W.T.); d05628007@ntu.edu.tw (H.-W.H.); 2 Institute of Veterinary Clinical Science, School of Veterinary Medicine, National Taiwan University, Taipei 106328, Taiwan; yajanelee@ntu.edu.tw; 3 Department of Internal Medicine, National Taiwan University Veterinary Hospital, Taipei 106319, Taiwan; 4 Center for Biotechnology, National Taiwan University, Taipei 106038, Taiwan

**Keywords:** chronic kidney disease, gut microbiota, probiotics, Lm pet treats, feline

## Abstract

**Simple Summary:**

Chronic kidney disease (CKD) is a vital issue waiting to be solved in the feline population. The efficacy of probiotics to prevent/alleviate CKD has been widely investigated in animal models. This pilot study combined a renal-protective functional *Lactobacillus* mixture (Lm) with pet feed as probiotic pet treats. CKD cats (stages 2 and 3) showed that kidney function and life quality were improved through modifying the composition of gut microbiota and metabolic patterns after administrating Lm pet treats daily for 8 weeks. This study clarified the possible mechanism of Lm in CKD cats and provided a possible novel way to treat cats with probiotics.

**Abstract:**

Microbiota-based strategies are a novel auxiliary therapeutic and preventative way of moderating chronic kidney disease (CKD). *Lactobacillus* mixture (Lm) was previously demonstrated to exert a renal-protective function in the CKD mice model. The efficacy of probiotics in pet foods is a relatively new area of study, and thus verifying the potential health benefits is necessary. This study evaluated the efficacy of Lm treats in feline CKD and elucidated the mechanisms underlying host-microbe interactions. CKD cats (2 and 3 stages) were administrated probiotic pet treats daily (10 g) for 8 weeks. The results demonstrated that during the eight weeks of Lm administration, creatinine was reduced or maintained in all cats with CKD. Similarly, gut-derived uremic toxin (GDUT), indoxyl sulfate (IS), were potential clinical significance in IS after Lm treatment (confidence intervals = 90%). The life quality of the cats also improved. Feline gut microbiome data, metabolic functional pathway, and renal function indicator analyses revealed the possible mechanisms involved in modulating CKD feline microbial composition. Further regulation of the microbial functions in amino acid metabolism after Lm administration contributed to downregulating deleterious GDUTs. The current study provides potential adjuvant therapeutic insights into probiotic pet foods or treats for pets with CKD.

## 1. Introduction

Chronic kidney disease (CKD) is defined as structural or functional abnormalities in the kidneys presenting for more than three months [1]. Decreased kidney clearance causes uremic toxins to accumulate in the body, thereby leading to damage to the kidneys, the cardiovascular system, the immune system, and the intestines [2,3]. In felines, the average prevalence of CKD is ~1–3% but increases to 80% in the geriatric feline population (>15 years old) [4,5]. Common symptoms of CKD in cats include weight and muscle loss, vomiting, anorexia/hyporexia (up to 92% of cats), constipation, proteinuria, non-regenerative anemia, and hypokalemia [1,6,7,8]. To date, there is no treatment without disadvantages and side effects for CKD [9]. CKD progression may be controlled by limiting protein intake, decreasing uremic toxin absorption, dialysis, or kidney transplantation [1,10,11]. Phosphorus level and the calcium:phosphorus (Ca:P) ratio in diet are critical factors to consider. Ideally, the dietary Ca:P ratio should be 1 or greater [7].

Indoxyl sulfate (IS), *p*-cresyl sulfate (PCS), and trimethylamine *N*-oxide (TMAO) are gut-derived uremic toxins (GDUTs) resulting from the breakdown of dietary protein by some proteolytic bacteria, such as *Escherichia coli*, *Clostridium difficile*, and *Shigella*, in the host intestine [10,12,13,14]. Increased circulating IS and PCS levels are negatively correlated with kidney function and strongly associated with a decline in the estimated glomerular filtration rate [15,16]. Thus, microbiota-based strategies could provide a potential auxiliary therapeutic and preventative method for CKD.

The efficacy of probiotics to prevent/alleviate CKD has been intensively investigated in animal models. Probiotics not only enhance the homeostasis in the intestine but also reduce the production or retention of uremic toxins. *Lactobacillus acidophilus* NT decreased urinary protein excretion and urea nitrogen, IS, and PCS in the serum of 5/6 nephrectomy mice with mitigating systemic inflammation and kidney sclerosis [17]. In a cisplatin-induced CKD Lanyu pig model, the probiotic mix downregulated IS levels in serum, and reduced fibrosis and oxidative stress in the kidney by shifting the composition of gut microbiota toward the normal control group [18]. In clinical studies, CKD dogs and cats also showed improved kidney function after treatment with probiotics [19,20]. However, most studies lacked in-depth mechanism investigation.

In our previous study, a *Lactobacillus* mixture (Lm, *Lacticaseibacillus paracasei* subsp. *paracasei* MFM 18 and *Lactiplantibacillus plantarum* subsp. *plantarum* MFM 30-3), isolated from traditional fermented milk, exerted kidney-protective effects in a CKD mice model [9] and human clinical trial [21]. The possible mechanism of Lm was also elucidated, which involved Lm-mediated interconnection and modulation of microbial composition, metabolic reactions, and metabolite profiles.

Treating pets with probiotics in powder, capsule, or tablet form is challenging [22], so combining probiotics with feed or pet treats might provide a potential solution. However, certain factors, such as microorganism characteristics (thermal resistance, oxygen tolerance, acid, and bile resistance), processing conditions (including time, temperature, pressure, moisture, water activity, and pH), application method, and packaging and storage conditions, affect probiotic survivability, which further influences their efficacy [23]. The efficacy of probiotics in pet foods is a new field of study, and inventions in the form of new application strategies, effective strain selection, and verification of the potential health benefits are necessary to ensure the product’s effectiveness [24]. Thus, the present study evaluated the efficacy of Lm pet treats in feline CKD and elucidated the mechanisms underlying host-microbe interactions. This study clarified the possible mechanism of Lm in CKD cats and provided a possible novel way to treat cats with probiotics.

## 2. Materials and Methods

### 2.1. Bacterial Strains

Lm consisted of *L*. *paracasei* subsp. *paracasei* MFM 18 and *L*. *plantarum* subsp. *plantarum* MFM 30-3 isolated from Mongolian fermented milk (MFM) in our lab, in a ratio of 1:1. The Lm culture conditions were as previously described [9]. The freeze-dried Lm powder was produced by Grape King Bio, Ltd. (Taoyuan, Taiwan) with microcrystalline α-cellulose, magnesium stearate, silicon dioxide, and oligofructose as an excipient for the production of Lm pet treats. The total bacterial count in the Lm powder was 1.07 × 10^11^ CFU/g.

### 2.2. Preparation of Lm Pet Treats

The Lm powder was mixed with chicken oil and fish oil at 37 °C. Then, 1% of the Lm powder was spread-coated at a low temperature (37 °C) on the commercial pet feed made by Withpet Inc. (Taoyuan, Taiwan) as Lm pet treats. Three different flavors of Lm pet treats were produced including chicken and fish (CA), fish and mutton (CB), and chicken (CC). The comprehensive ingredient of Lm pet treats is shown in Table 1 and Table S1.

### 2.3. Safety and Stability Testing of Lm Pet Treats

Lm pet treats were stored at room temperature. Harmful residue analysis and chemical stability were analyzed by Eurofins Food Testing Taiwan Ltd. (Kaohsiung, Taiwan). The pathogenic bacteria test was performed by the National Animal Industry Foundation (Taipei, Taiwan). Lm pet treats were homogenized with sodium chloride liquid every 2 weeks and the lactic acid bacteria count was evaluated on lactobacilli MRS agar (Neogen Corporation, Lansing, MI, USA).

### 2.4. Clinical CKD Cat Trial: A Pilot Study

This single-arm pilot study was conducted at the National Taiwan University Veterinary Hospital, Taiwan, from August to November 2021. The study was approved by the Institutional Animal Care and Use Committee of National Taiwan University (IACUC approval no: NTU-110-EL-00042). All owners signed an informed consent form before allowing their cats to participate in the study.

**Figure 1:**
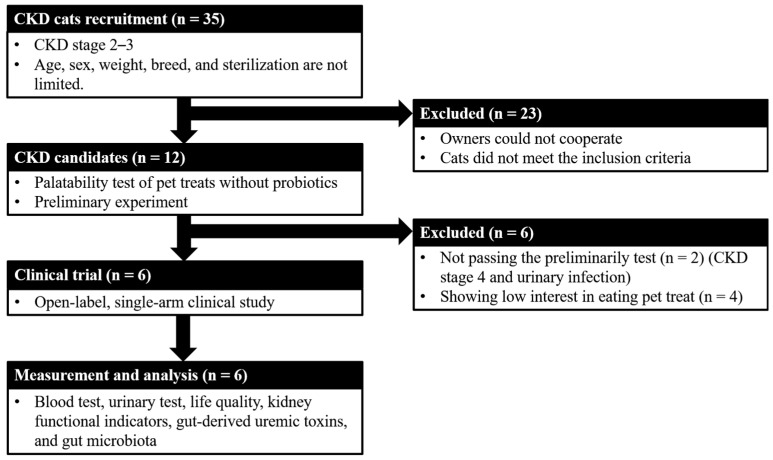
The study design.

The study design is shown in Figure 1. There were no limitations on the cats’ age, sex, breed, weight, and sterilization, but they were required to be CKD stages 2–3 and with 1.6–5.0 mg/dL creatinine or 18–38 μg/dL symmetric dimethylarginine (SDMA) [24] and meet one of the following conditions for at least 3 months: abnormal urinary test {urine specific gravity > 1.035; persistent renal proteinuria [urine protein/urine creatinine ratio (UPC) > 0.4]}, or subclinical symptoms (e.g., polyuria, polydipsia, and dehydration). Cats were excluded if they had acute kidney disease, acute worsening azotemia, diabetes, hyperthyroidism, urinary tract infection, or other nonrenal diseases (e.g., cardiac, hepatic, gastrointestinal, neoplastic diseases, or infection). Additionally, if cats were administered antibiotics 2 weeks before the beginning of the trial, they were also excluded.

This study involved 35 cats with CKD that were comprehensively evaluated to obtain previous clinical measurements, dietary and medical histories, and availability of clinical samples. Their owners provided consent for them to participate in this study. Their CKD stage and thyroxine levels were confirmed to ensure their suitability for study participation. Of the 12 cats with CKD enrolled in this clinical study, 6 completed the study and 6 dropped out due to unexpected complications including palatability of pet treats and a urinary tract infection, which may influence the evaluation of kidney function. The ratio of male to female cats was 1:1, with a median age of 13.0 (range 8–19 years) and a median weight of 4.41 kg (range 3.09–6.30 kg). One cat was CKD stage 2 and five were stage 3 (Table S2).

The cats were fed one bag of treats (10 g) to replace their original pet feed (10 g) by their owner daily for 8 weeks. Each bag of probiotic treats contained 2.79–3.93 × 10^8^ CFU/cat/day of Lm. The cats maintained regular CKD therapy and their original dietary habits at the time of enrollment and during the study period. After the whole experiment, owners answered a questionnaire about the life quality of the tested cats.

### 2.5. Biochemical Measurements

Biochemical analyses of blood and urine were conducted by the veterinary hospital. Creatinine, blood urea nitrogen (BUN), complete blood count, and ions in blood were measured by a blood chemistry analyzer. Urinary tests were also performed using the urine analyzer, including UPC and specific gravity.

### 2.6. Uremic Toxin Analysis

The gut-derived uremic toxins, including IS, PCS, TMAO, and phenyl sulfate (PS), were determined in plasma. Briefly, plasma (50 µL) was mixed with 50 µL of internal control (1000 ng/mL of PCS-d7, IS-d4, PS-13C6, and 100 ng/mL TMAO-d9) and 400 µL of acetonitrile, then centrifuged at 15,000× *g* rpm for 15 min at 4 °C. The supernatant (200 µL) was vacuum-concentrated and dissolved in 200 µL of 20% acetonitrile. The uremic toxins were measured by an LC-MS/MS system (TripleQuad5500, ABSCIEX, Framingham, MA, USA) with an ACQUITY UPLC BEH C18 Column (2.1 × 150 mm, 1.7 μm, waters). The mobile phase A was 1 mL methanol in 1000 mL ddH_2_O, and the mobile phase B was 10 mM ammonium acetate in 1000 mL acetonitrile. The eluting gradient was as follows: 0.0→3.0 min (10%→95% B); 3.0→4.0 min (95% B); 4.0→4.1 min (95→10% B); 4.1→6.0 min (0% B). The injection volume was 5 μL and the flow rate was 0.3 mL/min. The nebulizer gas pressure and drying gas pressure were both 55 psi, and the drying gas temperature was 550 °C in the positive electrospray ionization mode and negative electrospray ionization mode. The capillary voltage was 5.5 kV and −4.5 kV in positive and negative electrospray ionization mode, respectively. The model parameters of the multiple reaction monitoring of target uremic toxins are shown in Table S3.

### 2.7. Microbiota Analysis in Feline Feces

Fecal samples were collected at week 0 and 8, respectively. The fecal genomic DNA samples were extracted and stored at −20 °C. The DNA concentration was measured by a Qubit 4.0 Fluorometer (Thermo Scientific, Rockford, IL, USA) before third-generation sequencing assays were performed by BIOTOOLS Co., Ltd. (Taipei, Taiwan). The 16S whole-length sequencing was amplified by universal primers 27F: 5′-AGRGTTYGATYMTGGCTCAG-3′, and 1492R: 5′ RGYTACCTTGTTACGACTT-3′. In this study, Hifi reads with read quality (RQ) > 30 were retained and the DADA2 package (dada2_1.20) in R software was used to denoise. The reads after denoising are called ASVs (amplicon sequence variants) and one ASV was regarded as one species cluster. QIIME2 (v2021.4; http://qiime2.org/ (accessed on 18 November 2020)) was used to process and analyze the representative sequencing of the same ASVs. The NCBI 16S ribosomal RNA database (July 2020) was used to identify the taxonomy classification. The observed species, Shannon-Wiener diversity index, and Pielou’s evenness were analyzed through QIIME2 as the α diversity indexes. Beta diversity used principal coordinate analysis (PCoA) to compare the differences between microbiota composition before and after administrating Lm pet treats.

Specific bacteria, including *Bifidobacterium*, *Lactobacillus*, and *Enterobacteriaceae*, were quantified by qPCR. Each reaction included 5 μL 2× KAPA SYBR FAST qPCR Master Mix (Kapa Biosystems, Wilmington, MA, USA), 0.2 μL 10 μM forward primer, 0.2 μL 10 μM reverse primer (Table S4), 2 μL template DNA, and 2.6 μL ddH_2_O.

The PICRUSt (Phylogenetic Investigation of Communities by Reconstruction of Unobserved States) and the Kyoto Encyclopedia of Genes and Genomes (KEGG) database were applied to compare the gene information to predict metabolic function changes. In Spearman correlation analysis, kidney function indexes (BUN and creatinine), uremic toxins (IS, PS, TMAO, and PCS), and KEGG level 3 pathways were correlated with the bacterial biomarkers in the third-generation sequencing analysis.

### 2.8. Statistical Analysis

Lactic acid bacteria storage stability of Lm pet treats was shown as mean ± SD, and analyzed through one-way ANOVA by GraphPad Prism v9.3.1 (GraphPad Software Inc., Boston, MA, USA). All data of the clinical study were presented as mean ± SEM. Based on the results of the Shapiro–Wilk normality test, the statistical analyses were performed by the Wilcoxon signed-rank test or ratio paired *t*-test by GraphPad Prism v9.3.1 and Statistical Analysis System v9.4 (SAS Institute Inc., Cary, NC, USA). Considering the limited sample size in this pilot study and the resulting limitations in statistical power, it is possible that the effects of the Lm intervention may be underestimated. The treatment effect of the Lm intervention in clinical measurements was assessed in conjunction with 90% confidence intervals (CIs) to elucidate its clinical significance [25,26].

## 3. Results

### 3.1. Safety and Stability of Lm Pet Treats

**Figure 2:**
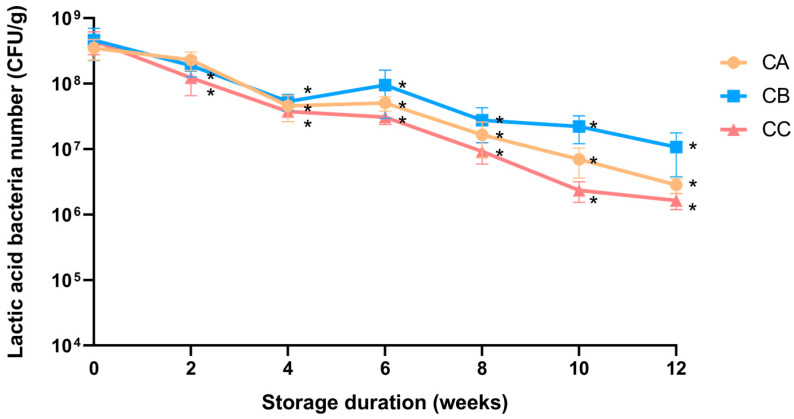
Lactic acid bacteria storage stability in Lm pet treats (50 g of probiotic powder per 5 kg of food). The data are presented as mean ± SD (n = 3). CA: chicken + fish; CB: fish + mutton; CC: chicken. * *p* < 0.05 indicates a significant difference compared to week 0.

The chemical and microbial analyses of the Lm pet treats showed no evidence of pathogenic bacteria or harmful residues, therefore the treats met all the relevant regulatory standards (Table S5). When stored at room temperature, there were no distinct changes in moisture, acid value, and peroxide value (POV) of the Lm pet treats (Table S6) after 8 weeks of storage. The average lactic acid bacteria count of the three flavors of Lm pet treats declined gradually from 4.3 × 10^8^ CFU/g to 5.4 × 10^6^ CFU/g after 12 weeks of storage (Figure 2). The effective dose of Lm in CKD cats (3–5 kg) was determined as 2.79–3.93 × 10^8^ CFU/cat/day [9,27]. Therefore, each cat weighing less than 5 kg should be given 10 g of Lm pet treats daily to receive the effective probiotic dosage. For cats weighing over 5 kg, the quantities of Lm pet treats were individually calculated to ensure they met the required probiotic intake. In our study, one cat weighed 6.3 kg and therefore required a daily intake of 12 g of Lm pet treats. The Lm pet treats were produced monthly and sent to owners directly to maintain the Lm viable dose.

### 3.2. Effect of Lm Pet Treats on Life Quality and Kidney Function in CKD Cats

**Figure 3:**
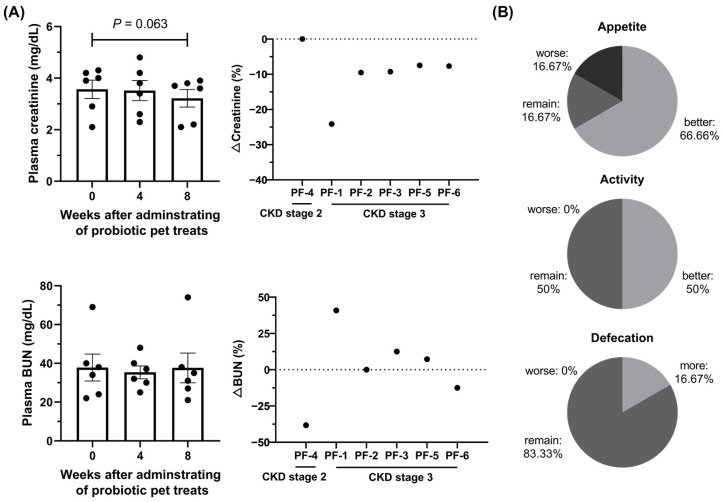
Effects of Lm pet treats on CKD cats. (**A**) Plasma value and the fold change of kidney function indicators. (**B**) Life quality of CKD cats. The data are presented as mean ± SEM (n = 6). Statistical analysis was performed using the Wilcoxon signed-rank test. PF: pet food group.

Blood and urine biochemical parameters were maintained during the administration of Lm treats, except serum phosphate, which was significantly elevated (*p* < 0.05) after taking Lm treats. The body weights of tested cats also remained stable (Table S7), indicating no adverse effects of Lm in cats with CKD (Table S8). Creatinine, a key indicator of kidney function, showed each measurement shifted towards a lower distribution (90% of CIs) with a *p*-value = 0.06 after 8 weeks of Lm treatment (Table 2), with all cats experiencing a reduction in creatinine, signifying a potential alleviatory effect of Lm treats on CKD progression (Figure 3A). BUN levels were also reduced or maintained in 50% of the cats. The kidney function indicators showed improvement for PF-1, who progressed from the third to the second stage of CKD (Table S9). The other subjects maintained their respective CKD stages.

After two months of Lm treatment, 66.67% (4/6 cats) of CKD cats had a better appetite with 100% (6/6 cats) of cats improving/maintaining their activity. One cat had a higher defecation frequency, and the others sustained the frequency (83.33%) (Figure 3B). Feedback from cat owners also reported that feline stool shape and color improved after the Lm treatment.

### 3.3. Effect of GDUTs in Plasma after the Administration of Lm Treats

**Figure 4:**
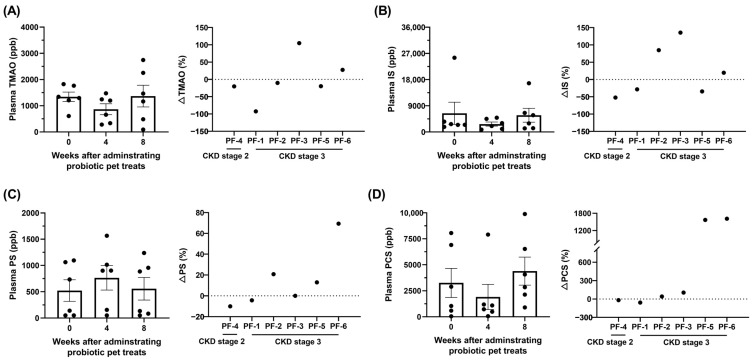
Plasma value and the fold change of uremic toxins after administrating 8 weeks of Lm pet treats. (**A**) Trimethylamine-*N*-oxide (TMAO), (**B**) indoxyl sulfate (IS), (**C**) phenyl sulfate (PS), and (**D**) *p*-cresyl sulfate (PCS). The data are presented as mean ± SEM (n = 6). Statistical analysis was performed using the Wilcoxon signed-rank test. PF: pet food group.

Although plasma TMAO, IS, PCS, and PS were not significantly different between groups (*p* > 0.05), GDUTs were further evaluated based on CIs (90%) to assess the treatment effect. The CIs in the present study suggest potential clinical significance in IS (Table 2) after 4 weeks of Lm treatment. A comparison of the percentage changes in individual cats, 66%, 50%, and 50% of the CKD cats decreased or maintained their plasma levels of TMAO, IS, and PCS, respectively, after 8 weeks of Lm treatment (Figure 4).

### 3.4. Lm Pet Treats Modified Fecal Microbiota of CKD Cats

**Figure 5:**
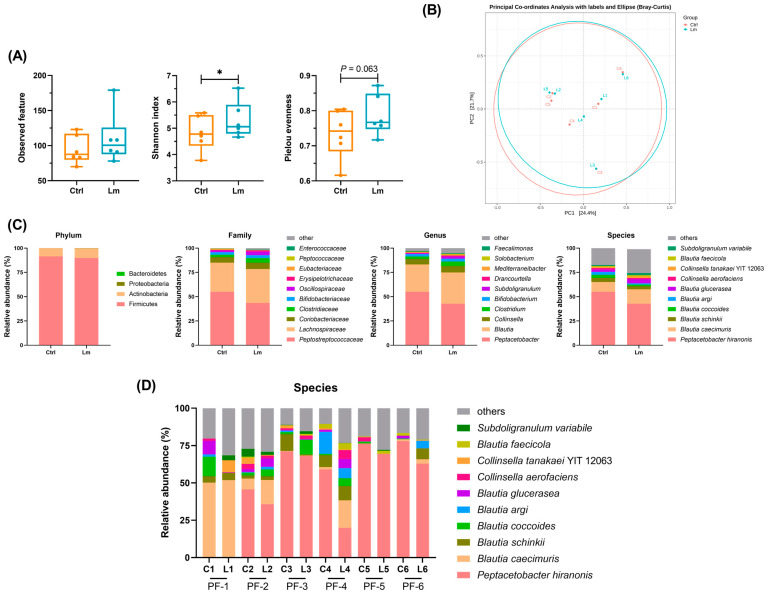
Fecal microbiota analyses of CKD cats. (**A**) Differences before and after the trial in fecal microbiota in different CKD cats. The results are shown as quartiles with minimum and maximum (n = 6). Statistical analysis was performed using the Wilcoxon signed-rank test. * *p* < 0.05 indicates a significant difference compared with week 0. (**B**) Alpha diversity. (**C**) Beta diversity. (**D**) Taxonomic shifts in species level. Each dot represents a cat. Letters C and Ctrl represent the fecal sample before administrating Lm; letters L and Lm represent the fecal sample after administrating Lm.

We used 16S full-length sequencing to explore the link between Lm treatment and the gut microbiota composition, showing that the Shannon index and alpha diversity index significantly increased after the Lm treatment (*p* < 0.05), while the Pielou evenness increased (*p* = 0.06) (Figure 5A). Conversely, beta diversity in the PCoA plot showed only a slight shift, indicating 24.4% and 21.7% of the total gut microbiota composition in PC1 and PC2, respectively (Figure 5B). These findings indicate that Lm increased the richness of microbial species that were phylogenetically similar, enhanced evenness among these species, and simultaneously maintained the core gut microbiome composition in felines.

Regarding the gut microbial configuration before and after administrating Lm treats, there were four dominant phyla including Firmicutes (~90%), Actinobacteria (~10%), Proteobacteria, and Bacteroidetes (Figure 5C), and ten prominent families, with *Peptostreptococcaceae* being the most dominant family (from 55.0% to 43.5%), followed by *Lachnospiraceae* (from 30.0% to 35.0%), *Coriobacteriaceae* (from 5.8% to 6.62%), *Clostridiaceae* (from 2.4% to 4.6%), *Bifidobacteriaceae* (from 2.6% to 3.0%), *Oscillospiraceae* (from 1.7% to 3.7%), *Erysipelotrichaceae* (from 0.7% to 1.2%), *Eubacteriaceae* (from 0.7% to 0.3%), *Pepcococcaceae* (from 0.6% to 0.2%), and *Enterococaceae* (from 0.0% to 0.5%). *Peptacetobacter* (from 55.0% to 42.7%) was the most abundant at the genus level, followed by *Blautia* (from 28.2% to 32.3%), *Collinsella* (from 5.7% to 6.6%), *Clostridium* (from 2.4% to 4.6%), *Bifidobacterium* (from 2.6% to 3.0%), *Subdoligranulum* (from 0.9% to 2.1%), *Drancourtella* (from 0.5% to 1.1%), *Mediterraneibacter* (from 0.7% to 0.9%), *Solobacterium* (from 0.5% to 0.8%) and *Faecalimonas* (remaining 0.5%) (Figure 5C).

A total of 121 bacterial species were identified in the feline gut microbiome and the top ten were *Peptacetobacter hiranonis* (from 55.0% to 42.7%), *Blautia caecimuris* (from 10.1% to 14.9%), *Blautia schinkii* (from 4.3% to 3.3%), *Blautia coccoides* (from 3.1% to 1.6%), *Blautia argi* (from 3.0% to 1.3%), *Blautia glucerasea* (from 2.5% to 4.2%), *Collinsella aerofaciens* (from 1.5% to 1.3%), *Collinsella tanakaei* (from 1.1% to 1.7%), *Blautia faecicola* (from 1.0% to 2.3%), and *Subdoligranulum variabile* (from 0.9% to 2.1%) (Figure 5C).

**Figure 6:**
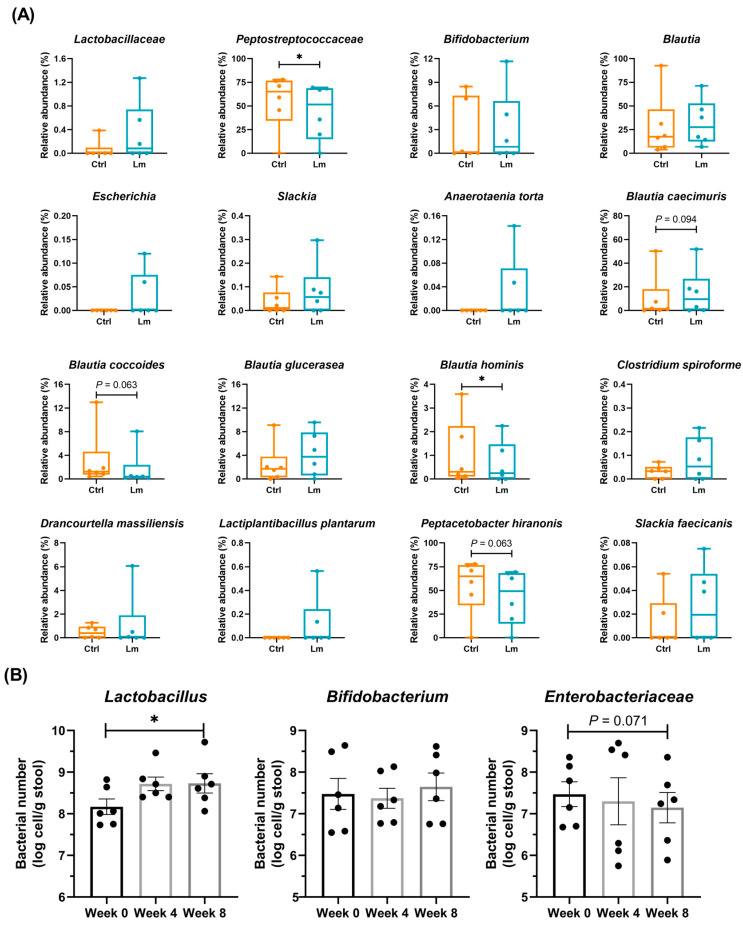
(**A**) Relative abundance of specific bacteria in fecal microbiota. The results are shown as quartiles with minimum and maximum (n = 6). Group Ctrl and Lm represent the samples before and after administrating probiotics pet treats, respectively. (**B**) The specific bacterium number per gram of CKD cats’ feces. The results are presented as mean ± SEM (n = 6). Each dot represents one cat. Statistical analysis was performed using Wilcoxon signed-rank test in relative abundance analyses, and ratio paired *t*-test in qPCR analyses. * *p* < 0.05.

There were also variations in the individual fecal microbiomes at the species level (Figure 5D), for example, the PF-1 cat had a high proportion of *Blautia*, while the other 5 cats had more *P. hiranonis*. However, they showed a similar change in fecal microbiota after treatment with Lm pet treats (Figure 6A). *Peptostreptococcaeae* significantly reduced (*p* < 0.05), while *Lactobacillaceae* and *Bifidobacterium* increased from week 0 to week 8. At the species level, *Blautia hominis* (*p* < 0.05), *B. coccoides* (*p* = 0.063), and *P. hiranonis* (*p* = 0.063) were reduced but the genus *Blautia* increased after the trial. Besides, one of the Lm bacterial strains (*L. plantarum*) was detected in two cats after the clinical trial.

Bacteria related to CKD in fecal microbiota were quantified by qPCR (Figure 6B) showing that *Lactobacillus* and *Enterobacteriaceae* were significantly upregulated (*p* < 0.05) and downregulated (*p* = 0.071) after 8 weeks of Lm pet treats administration, respectively.

### 3.5. Lm Pet Treats Altered Gut Microbial Function

**Figure 7:**
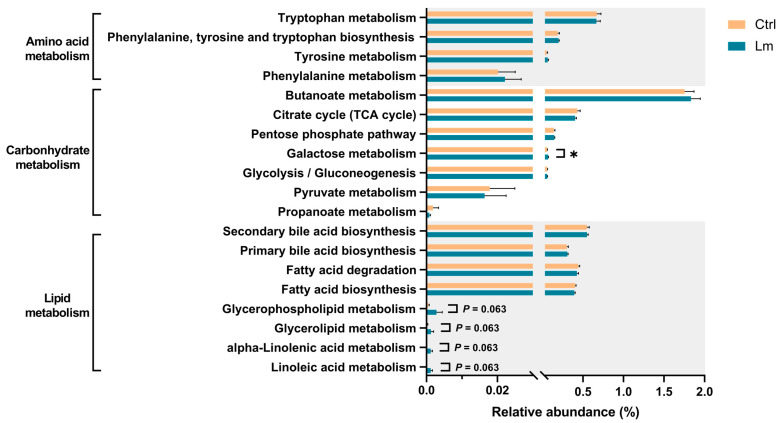
Comparison of the relative abundance of the PICRUSt functional prediction of the fecal microbiota between before/after trial. The results are presented as mean ± SEM (n = 6). Group Ctrl and Lm represent the samples before and after administrating probiotics pet treats, respectively. Distinct gene categories were selected in KEGG pathway level 3. Statistical analysis was performed using Wilcoxon matched-pairs signed-rank test. * *p* < 0.05.

Genetic functional prediction was performed (Figure 7) revealing that the pathways related to producing uremic toxins, including “Tyrosine metabolism (ko00350),” “Phenylalanine metabolism (ko00360)”, “Tryptophan metabolism (ko00380)”, and “Phenylalanine, tyrosine and tryptophan biosynthesis (ko00400)”, were not different after Lm treatment, whereas the carbohydrate-related pathway, “Galactose metabolism (ko00052)” was significantly higher (*p* < 0.05). After the clinical trial, the lipid metabolism pathways “Glycerolipid metabolism (ko00561)”, “Glycerophospholipid metabolism (ko00564)”, “Linoleic acid metabolism (ko00591)”, and “Alpha-linolenic acid metabolism (ko00592)” were upregulated (*p* < 0.1).

**Figure 8:**
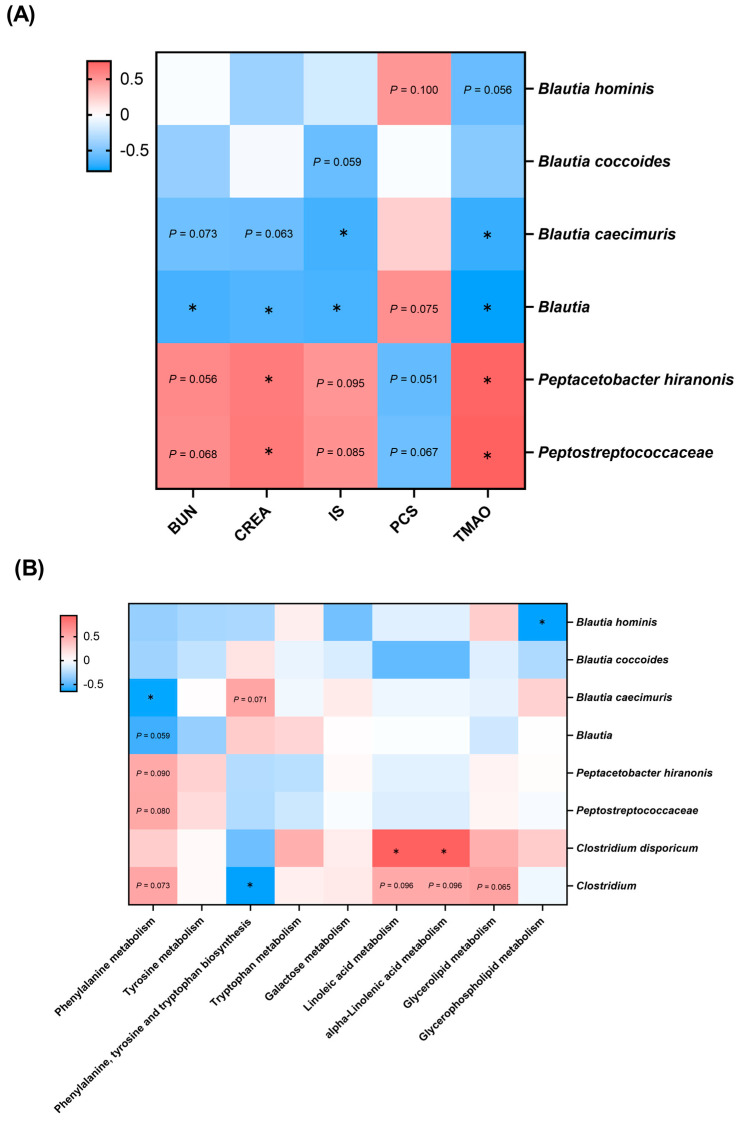
Spearman correlation analysis of biomarkers and (**A**) risk factors of CKD progression and (**B**) KEGG pathways. * *p* < 0.05. BUN: blood urea nitrogen; CREA: creatinine; IS: indoxyl sulfate; PCS: *p*-cresyl sulfate; PS: phenyl sulfate; TMAO: trimethylamine *N*-oxide.

A Spearman’s correlation network was first constructed between eight bacterial species and 5 CKD risk factors that were significantly different before and after administration of the Lm treats (Figure 8A). All bacteria belonging to the genus *Blautia* were negatively correlated with BUN and creatinine, particularly the genus *Blautia* (*p* < 0.05). In uremic toxins analyses, *B. caecimuris* and *Blautia* were significantly negatively correlated with IS and TMAO (*p* < 0.05), whereas *Peptostreptococcaceae* and *P. hiranonis* demonstrated a significant positive correlation with kidney function indicators and uremic toxins, especially creatinine and TMAO (*p* < 0.05). The correlation network between bacterial species and KEGG pathways revealed two and three positive and negative relationships with KEGG level 3 pathways, respectively (*p* < 0.05, Figure 8B). *B. caecimuris* and *Clostridium* were negatively correlated with phenylalanine metabolism and phenylalanine, tyrosine and tryptophan biosynthesis pathways, respectively (Figure 8B).

## 4. Discussion

In the present study, a novel low-temperature oil-spreading approach was developed to produce pet food coated with *Lactobacillus* mix (Lm, *L. plantarum* subsp. *plantarum* MFM 30−3 and *L. paracasei* subsp. *paracasei* MFM 18). The produced Lm pet treats were chemically and bacterially stable with a 4-month shelf life at room temperature. Pet food production designed to ensure food safety and to extend shelf-life negatively impacts the survival of probiotics [24]. Additional verification of the potential health benefits is crucial to ensure the efficacy of probiotics in pet foods. Although there was no significant difference in plasma indicators and GDUTs before and after treating with Lm probiotic treats (due to the limited sample size), we still found the potential CKD alleviatory effect of Lm probiotic treats in this open-label, single-arm pilot study of cats with stage 2–3 CKD. The clinical tendency for downregulation of harmful GDUT (IS) and CKD plasma indicators (creatinine and BUN) (confidence intervals = 90%) and improved life quality (appetite, activity, and defecation frequency) of CKD cats were observed after two months of the Lm pet treats intervention. This is in line with our previous studies using an adenine-induced CKD mouse model [9] and CKD patient clinical trial [21]. This finding provides evidence that Lm probiotics have the potential to be applied with various matrices without negatively impacting their health benefits. Statistical significance indicates the reliability of the study results, which is dependent on the study’s sample size [28], whereas clinical significance reflects its impact on clinical practice, which emphasizes the estimated effect size and its precision (such as confidence interval) [29]. To evaluate the actual treatment effect of Lm pet treats on CKD cats, the clinical significance was interpreted in this study through 90% confidence intervals and *p* < 0.1 [26,30].

Intensive studies have shown numerous outcomes to disclose the physiological functions of probiotics in human patients with CKD, including reduced uremic toxins and related precursors, modulation of gut microbiota, regulation of immune capacity, protection of the gastrointestinal tract, and improved gastrointestinal symptoms [31,32,33,34,35], but few studies have evaluated the effect of probiotics and probiotic pet food in feline CKD. Serum creatinine is one of the main evaluation indicators because it is the major parameter to calculate the estimated glomerular filtration rate (eGFR) to measure the stability of renal function [36]. Maintenance or reduction of plasma creatinine levels in all of the tested cats after the administration of Lm pet treats suggests a potential moderating effect on CKD in felines. A previous clinical study with CKD stage 2–4 cats showed that the BUN and creatinine levels of most cats decreased after taking synbiotics for two months [18], but this effect was not observed in the CKD counterparts with prebiotics that were mixed with or sprinkled onto the cat food.

The improved life quality of CKD cats, including appetite, activity, and defecation frequency, was also observed after two months of Lm treatment. Cats and patients with CKD have an increased risk of constipation, which would further impact their life quality [8,37,38]. Constipation may lead to enhanced fermentation of unmetabolized amino acids and peptides in the colon, resulting in the generation and absorption of more uremic toxins precursors [9,39]. Thus, a higher frequency of defecation and moister and softer feces would promote body waste discharge rather than accumulation. Therefore, Lm pet treats could be a novel way to supplement probiotics and offer potential benefits in terms of life quality and kidney function in cats with CKD.

Gut dysbiosis in patients with CKD contributes to deteriorating CKD progression [10,40,41,42]. Feline fecal bacteria diversity and abundance were restored with the Lm pet treats intervention. Most bacterial taxa that showed lower abundances after the Lm treatment belonged to proteolytic families (*Peptostreptococcaceae* and *Enterobacteriaceae*). *Peptostreptococcaceae* and *Enterobacteriaceae*, which possess GDUT precursor-producing enzymes contributing to indole, phenol, and TMAO production in humans, were more abundant in CKD patients than in healthy participants [43,44,45]. The correlation analysis is consistent with the findings of the feline gut microbiome. *Peptostreptococcaceae* was positively correlated to kidney function indicators and uremic toxins. The beneficial bacteria, *Lactobacillus*, in feline feces was elevated after administrating Lm pet treats. *Lactobacillaceae* (especially *Lactobacillus*), which are butyric acid-producing bacteria, are important for intestinal homeostasis [46,47,48]. Additionally, after consuming probiotic pet treats for 8 weeks, *L. plantarum* was detected in the feces of 2 tested cats (Figure 6A), indicating that Lm could be preserved in the intestine. The abundance of Lm strains may affect its efficacy in improving gut-derived metabolites and kidney function. However, further investigation is necessary to identify the factors impacting probiotic colonization.

Interestingly, the *Blautia* species demonstrated different changes in abundance in CKD cats after administrating Lm pet treats, but they were all negatively correlated with renal function indicators and GDUT. The less abundant genus, *Blautia,* has been reported in the gut microbiota of CKD patients and chronic renal failure (CRF) rats [44,49], and it is also negatively correlated with plasma uremic toxins [50]. However, *Blautia* was rich in patients with CKD stage 5 [44], suggesting that different strains of *Blautia* might cause different effects. It is also worth noting the effect of diet on gut microbiota, with the higher abundance of *Blautia* and *Peptostreptococcaceae* in the feline fecal microbiota associated with consuming kibbled meals and canned food, respectively [3]. This might explain why there was a difference in gut microbial composition between PF-1 and the other cats.

Alteration of gut microbial composition was associated with significant changes in KEGG microbial functions, with the phenylalanine pathway being most related to the CKD alleviatory effect of Lm pet treats. Abnormal phenylalanine metabolism has been detected in patients with diabetic kidney disease [51]. However, few studies have exposed the effect of probiotics on downstream microbial functions in patients and cats with CKD. The analyses of feline gut microbiota, KEGG microbial functions, and renal function indicators clarified the possible downstream mechanisms of the CKD-alleviating effects of Lm pet treats involving the downstream functional phenylalanine pathway.

## 5. Conclusions

Lm pet treats may offer a potential supportive option for mitigating the progression of CKD, with a possibility of improving the quality of life in cats with CKD. Administration of Lm pet treats modulated feline microbiota (*Peptostreptococcaceae*, *Lactobacillus, Blautia,* and *Enterobacteriaceae*), further regulating microbial functions involved in phenylalanine metabolism, contributing to downregulating deleterious IS. The abundance of Lm strains may influence their efficacy in improving gut-derived metabolites and kidney function. Although large-scale clinical studies are necessary to verify this finding, the current study provides potential adjuvant therapeutic insights into probiotic pet foods or treats for pets with CKD. To the best of our knowledge, this study is the first to directly evaluate CKD-alleviating efficacy in pet treats.

## 6. Limitation of the Study

The study sample size was restricted by the willingness of cat owners, the palatability of the pet treats, and rigorous candidate screening. The different diets used could also have caused inconsistent changes in microbiota composition, thus impacting achievement of statistical significance. Large-scale prospective longitudinal clinical studies are needed to confirm this finding in the future.

## Figures and Tables

**Table 1:** Nutrient composition of Lm pet treats.

	CA	CB	CC
Energy (kcal/100 g food)	411.0	413.9	425.7
Carbohydrates (%)	41.7	41.9	41.9
Crude protein (%)	33.6	33.0	31.9
Crude fat (%)	12.2	12.7	14.5
Crude fiber (%)	3.50	3.50	3.2
Sodium (Na) (mg/100 g food)	413.0	511.5	346.4
Phosphate (P) (mg/100 g food)	1210	1250	965
Ca/P	1.56	1.54	1.66
Ash (%)	7.4	7.7	6.4

CA: chicken + fish; CB: fish + mutton; CC: chicken.

**Table 2:** Kidney function indicators and uremic toxins in plasma of CKD cats during the trial.

Indicators	Before Lm Intervention	During Lm Intervention	After Lm Intervention	*p* Value	
	Week 0	Week 4	Week 8	Week 0 vs. 4	Week 0 vs. 8
BUN	37.83 (23.91–51.76)	35.33 (28.64–42.03)	37.67 (22.22–51.12)	0.851	0.906
creatinine	3.57 (2.85–4.29)	3.52 (2.73–4.30)	3.22 (2.53–3.90)	0.742	0.063
TMAO	1340.17 (977.60–1702.74)	865.95 (447.43–1284.48)	1367.73 (533.86–2201.60)	0.100	0.475
PS	522.05 (109.72–934.38)	764.80 (296.23–1233.37)	557.97 (124.62–991.31)	0.287	0.276
IS	6410.50 (−1306.75–14,127.75)	2727.87 (1304.41–4151.32)	5766.97 (977.79–10,554.14)	0.313	>0.999
PCS	3259.52 (439.16–6077.87)	1914.03 (−524.98–4353.04)	4394.73 (1681.35–7108.12)	0.156	0.202

Data were presented as mean (90% confidence intervals). Variables were tested using the ratio paired *t*-test or Wilcoxon signed-rank test.

## Data Availability

The authors confirm that the data supporting the findings of this study are available within the article and its Supplementary Materials. Raw data that support the findings of this study are available from the corresponding author upon reasonable request.

## Supplementary Materials

The following supporting information can be downloaded at: https://www.mdpi.com/article/10.3390/ani14040630/s1, Table S1. Comprehensive ingredient list of Lm pet treats; Table S2. Demographic characteristics of the study group; Table S3. Detection parameters of gut-derived uremic toxins in LC-MS/MS system; Table S4. qPCR primers for targeted microorganisms; Table S5. Harmful residue and pathogenic bacteria analyses of Lm pet treats; Table S6. Chemical stability analysis of Lm pet treats; Table S7. Weight changes in CKD cats throughout the trial; Table S8. Serum and urinary biochemical parameters of CKD cats throughout the trial; Table S9. Change of CKD stage of tested cats before and after the trial [52,53,54,55].

## Abbreviations

ASVs, amplicon sequence variants; BUN, blood urea nitrogen; CA, chicken and fish; CB, fish and mutton; CC, chicken; CKD, chronic kidney disease; CRF, chronic renal failure; ESRD, end-stage renal disease; IACUC, Institutional Animal Care and Use Committee of National Taiwan University; SDMA, symmetric dimethylarginine; IS, Indoxyl sulfate; KEGG, Kyoto Encyclopedia of Genes and Genomes; Lm, *Lactobacillus* mixture; MFM, Mongolian fermented milk; PCoA, principal coordinate analysis; PCS, *p*-cresyl sulfate; PF, pet food group; PICRUSt, Phylogenetic Investigation of Communities by Reconstruction of Unobserved States; POV, peroxide value; qPCR, real-time polymerase chain reaction; SCFA, short-chain fatty acid; TMAO, trimethylamine *N*-oxide; UPC, urine protein/urine creatinine ratio.

## References

[1] Polzin D.J. (2011). Chronic kidney disease in small animals. Vet. Clin. N. Am. Small Anim. Pract..

[2] Lu P.-H., Yu M.-C., Wei M.-J., Kuo K.-L. (2021). The Therapeutic strategies for uremic toxins control in chronic kidney disease. Toxins.

[3] Rosner M.H., Reis T., Husain-Syed F., Vanholder R., Hutchison C., Stenvinkel P., Blankestijn P.J., Cozzolino M., Juillard L., Kashani K. (2021). Classification of uremic toxins and their role in kidney failure. Clin. J. Am. Soc. Nephrol..

[4] Roura X. Risk Factors in Dogs and Cats for Development of Chronic Kidney Disease. https://www.iris-kidney.com/education/education/risk_factors.html.

[5] Chen H., Dunaevich A., Apfelbaum N., Kuzi S., Mazaki-Tovi M., Aroch I., Segev G. (2020). Acute on chronic kidney disease in cats: Etiology, clinical and clinicopathologic findings, prognostic markers, and outcome. J. Vet. Intern. Med..

[6] Bartges J.W. (2012). Chronic kidney disease in dogs and cats. Vet. Clin. N. Am. Small Anim. Pract..

[7] Parker V.J. (2021). Nutritional management for dogs and cats with chronic kidney disease. Vet. Clin. N. Am. Small Anim. Pract..

[8] Jones S.E., Quimby J.M., Summers S.C., Adams S.M., Caney S.M., Rudinsky A.J. (2022). Survey of defecation habits in apparently healthy and chronic kidney disease cats. J. Feline Med. Surg..

[9] Huang H., Li K., Lee Y., Chen M. (2021). Preventive effects of *Lactobacillus* mixture against chronic kidney disease progression through enhancement of beneficial bacteria and downregulation of gut-derived uremic toxins. J. Agric. Food Chem..

[10] Evenepoel P., Meijers B.K., Bammens B.R., Verbeke K. (2009). Uremic toxins originating from colonic microbial metabolism. Kidney Int..

[11] Koppe L., Mafra D., Fouque D. (2015). Probiotics and chronic kidney disease. Kidney Int..

[12] Lim Y.J., Sidor N.A., Tonial N.C., Che A., Urquhart B.L. (2021). Uremic toxins in the progression of chronic kidney disease and cardiovascular disease: Mechanisms and therapeutic targets. Toxins.

[13] Subramaniam S., Fletcher C. (2018). Trimethylamine *N*-oxide: Breathe new life. Br. J. Pharmacol..

[14] Carlström M., Moretti C.H., Weitzberg E., Lundberg J.O. (2020). Microbiota, diet and the generation of reactive nitrogen compounds. Free Radic. Biol. Med..

[15] Wu I.W., Hsu K.H., Lee C.C., Sun C.Y., Hsu H.J., Tsai C.J., Tzen C.Y., Wang Y.C., Lin C.Y., Wu M.S. (2011). *p*-Cresyl sulphate and indoxyl sulphate predict progression of chronic kidney disease. Nephrol. Dial. Transplant..

[16] Pretorius C.J., McWhinney B.C., Sipinkoski B., Johnson L.A., Rossi M., Campbell K.L., Ungerer J.P. (2013). Reference ranges and biological variation of free and total serum indoxyl- and *p*-cresyl sulphate measured with a rapid UPLC fluorescence detection method. Clin. Chim. Acta.

[17] Yoshifuji A., Wakino S., Irie J., Tajima T., Hasegawa K., Kanda T., Tokuyama H., Hayashi K., Itoh H. (2016). Gut *Lactobacillus* protects against the progression of renal damage by modulating the gut environment in rats. Nephrol. Dial. Transplant..

[18] Lee Y.-J., Li K.-Y., Wang P.J., Huang H.W., Chen M.J. (2020). Alleviating chronic kidney disease progression through modulating the critical genus of gut microbiota in a cisplatin-induced Lanyu pig model. J. Food Drug Anal..

[19] Palmquist R. (2006). A preliminary clinical evaluation of Kibow Biotics^®^, a probiotic agent, on feline azotemia. J. Am. Holistic Vet. Med. Assoc..

[20] Lippi I., Perondi F., Ceccherini G., Marchetti V., Guidi G. (2017). Effects of probiotic VSL#3 on glomerular filtration rate in dogs affected by chronic kidney disease: A pilot study. Can. Vet. J..

[21] Chan W.N., Ho D.R., Huang Y.C., Lin J.H., Liu Y.L., Chen M.J., Chen C.S. (2023). A pilot study of nephrogenic probiotics to further improve an already stabilized graft function after kidney transplantation. Transplant. Proc..

[22] Rishniw M., Wynn S.G. (2011). Azodyl, a synbiotic, fails to alter azotemia in cats with chronic kidney disease when sprinkled onto food. J. Feline Med. Surg..

[23] Chuang J.J., Huang Y.Y., Lo S.H., Hsu T.F., Huang W.Y., Huang S.L., Lin Y.S. (2017). Effects of pH on the shape of alginate particles and its release behavior. Int. J. Polym. Sci..

[24] Acuff H., Aldrich C.G. (2023). A Review of Application Strategies and Efficacy of Probiotics in Pet Food.

[25] International Renal Interest Society IRIS Staging of CKD (Modified 2023). http://www.iris-kidney.com/pdf/2_IRIS_Staging_of_CKD_2023.pdf.

[26] Ranganathan P., Pramesh C.S., Buyse M. (2015). Common pitfalls in statistical analysis: Clinical versus statistical significance. Perspect. Clin. Res..

[27] Nair A., Morsy M.A., Jacob S. (2018). Dose translation between laboratory animals and human in preclinical and clinical phases of drug development. Drug Dev. Res..

[28] Moore M.J., Goldstein D., Hamm J., Figer A., Hecht J.R., Gallinger S., Au H.J., Murawa P., Walde D., Wolff R.A. (2007). Erlotinib plus gemcitabine compared with gemcitabine alone in patients with advanced pancreatic cancer: A phase III trial of the National Cancer Institute of Canada Clinical Trials Group. J. Clin. Oncol..

[29] Schulz K.F., Altman D.G., Moher D., CONSORT Group (2010). CONSORT 2010 statement: Updated guidelines for reporting parallel group randomised trials. BMJ.

[30] Phillips M.R., Wykoff C.C., Thabane L., Bhandari M., Chaudhary V., Retina Evidence Trials InterNational Alliance (R.E.T.I.N.A.) Study Group (2022). The clinician’s guide to p values, confidence intervals, and magnitude of effects. Eye.

[31] Cosola C., Rocchetti M.T., di Bari I., Acquaviva P.M., Maranzano V., Corciulo S., Di Ciaula A., Di Palo D.M., La Forgia F.M., Fontana S. (2021). An innovative synbiotic formulation decreases free serum indoxyl sulfate, small intestine permeability and ameliorates gastrointestinal symptoms in a randomized pilot trial in stage IIIb-IV CKD patients. Toxins.

[32] Liu S., Liu H., Chen L., Liang S.S., Shi K., Meng W., Xue J., He Q., Jiang H. (2020). Effect of probiotics on the intestinal microbiota of hemodialysis patients: A randomized trial. Eur. J. Nutr..

[33] Mitrović M., Stanković-Popović V., Tolinački M., Golić N., Soković Bajić S., Veljović K., Nastasijević B., Soldatović I., Svorcan P., Dimković N. (2023). The impact of synbiotic treatment on the levels of gut-derived uremic toxins, inflammation, and gut microbiome of chronic kidney disease patients-a randomized trial. J. Renal Nutr..

[34] Wang I.K., Yen T.H., Hsieh P.S., Ho H.H., Kuo Y.W., Huang Y.Y., Kuo Y.L., Li C.Y., Lin H.C., Wang J.Y. (2021). Effect of a probiotic combination in an experimental mouse model and clinical patients with chronic kidney disease: A pilot study. Front. Nutr..

[35] Lim P.S., Wang H.F., Lee M.C., Chiu L.S., Wu M.Y., Chang W.C., Wu T.K. (2021). The Efficacy of *Lactobacillus*-containing probiotic supplementation in hemodialysis patients: A randomized, double-blind, placebo-controlled trial. J. Renal Nutr..

[36] Shahbaz H., Gupta M. Creatinine Clearance. StatPearls [Internet]. https://www.ncbi.nlm.nih.gov/books/NBK544228/2.

[37] Sumida K., Molnar M.Z., Potukuchi P.K., Thomas F., Lu J.L., Matsushita K., Yamagata K., Kalantar-Zadeh K., Kovesdy C.P. (2017). Constipation and incident CKD. J. Am. Soc. Nephrol..

[38] Ikee R., Sasaki N., Yasuda T., Fukazawa S. (2020). Chronic kidney disease, gut dysbiosis, and constipation: A burdensome triplet. Microorganisms.

[39] Sumida K., Yamagata K., Kovesdy C.P. (2019). Constipation in CKD. Kidney Int. Rep..

[40] Dieterich W., Schink M., Zopf Y. (2018). Microbiota in the gastrointestinal tract. Med. Sci..

[41] Targher G., Byrne C.D. (2017). Non-alcoholic fatty liver disease: An emerging driving force in chronic kidney disease. Nat. Rev. Nephrol..

[42] Jiang S., Xie S., Lv D., Wang P., He H., Zhang T., Zhou Y., Lin Q., Zhou H., Jiang J. (2017). Alteration of the gut microbiota in Chinese population with chronic kidney disease. Sci. Rep..

[43] Bermingham E.N., Young W., Butowski C.F., Moon C.D., Maclean P.H., Rosendale D., Cave N.J., Thomas D.G. (2018). The fecal microbiota in the domestic cat (*Felis catus*) is influenced by interactions between age and diet; a five year longitudinal study. Front. Microbiol..

[44] Cai H., Su S., Li Y., Zhu Z., Guo J., Zhu Y., Guo S., Qian D., Duan J. (2019). Danshen can interact with intestinal bacteria from normal and chronic renal failure rats. Biomed. Pharmacother..

[45] Bäckhed F. (2013). Meat-metabolizing bacteria in atherosclerosis. Nat. Med..

[46] Wong J., Piceno Y.M., DeSantis T.Z., Pahl M., Andersen G.L., Vaziri N.D. (2014). Expansion of urease- and uricase-containing, indole- and *p*-cresol-forming and contraction of short-chain fatty acid-producing intestinal microbiota in ESRD. Am. J. Nephrol..

[47] Sampaio-Maia B., Simões-Silva L., Pestana M., Araujo R., Soares-Silva I.J. (2016). The role of the gut microbiome on chronic kidney disease. Adv. Appl. Microbiol..

[48] Rukavina Mikusic N.L., Kouyoumdzian N.M., Choi M.R. (2020). Gut microbiota and chronic kidney disease: Evidences and mechanisms that mediate a new communication in the gastrointestinal-renal axis. Pfluegers Arch..

[49] Ren Z., Fan Y., Li A., Shen Q., Wu J., Ren L., Lu H., Ding S., Ren H., Liu C. (2020). Alterations of the human gut microbiome in chronic kidney disease. Adv. Sci..

[50] Pan L., Han P., Ma S., Peng R., Wang C., Kong W., Cong L., Fu J., Zhang Z., Yu H. (2020). Abnormal metabolism of gut microbiota reveals the possible molecular mechanism of nephropathy induced by hyperuricemia. Acta Pharm. Sin. B..

[51] Grabrucker S., Marizzoni M., Silajdžić E., Lopizzo N., Mombelli E., Nicolas S., Dohm-Hansen S., Scassellati C., Moretti D.V., Rosa M. (2023). Microbiota from Alzheimer’s patients induce deficits in cognition and hippocampal neurogenesis. Brain.

[52] Matsuki T., Watanabe K., Fujimoto J., Miyamoto Y., Takada T., Matsumoto K., Oyaizu H., Tanaka R. (2002). Development of 16S rRNA-gene-targeted group-specific primers for the detection and identification of predominant bacteria in human feces. Appl. Environ. Microbiol..

[53] Matsuda K., Tsuji H., Asahara T., Kado Y., Nomoto K. (2007). Sensitive quantitative detection of commensal bacteria by rRNA-targeted reverse transcription-PCR. Appl. Environ. Microbiol..

[54] Rinttilä T., Kassinen A., Malinen E., Krogius L., Palva A. (2004). Development of an extensive set of 16S rDNA-targeted primers for quantification of pathogenic and indigenous bacteria in faecal samples by real-time PCR. J. Appl. Microbiol..

[55] Kikuchi E., Miyamoto Y., Narushima S., Itoh K. (2002). Design of species-specific primers to identify 13 species of *Clostridium* harbored in human intestinal tracts. Microbiol. Immunol..

