# Supplementary material for: Investigating the Efficacy of Kidney-Protective Lactobacillus Mixture-Containing Pet Treats in Feline Chronic Kidney Disease and Its Possible Mechanism
Source: Animals (Basel). 2024 Feb 16;14(4):630. doi: 10.3390/ani14040630 (PMC10886156; doi:10.3390/ani14040630)
Supplement: Supplementary file 1 [file animals-14-00630-s001.zip › animals-2807310-supplementary.pdf]

Supplementary Materials

Table S1. Comprehensive ingredient list of Lm pet treats

	CA	CB	CC
**Ingredients**	Dried chicken, tapioca starch, fresh sea fish (cod, common dolphinfish), black beans, chicken fat, hydrolyzed peptides, molasses yeast, potato starch, dried egg yolk, Taiwan No. 57 dried sweet potato, coconut powder, functional cellulose, fish oil, sodium tripolyphosphate, choline chloride, yeast peptide, taurine, multivitamins (A, B complex, D, E), comprehensive amino acid chelated minerals (zinc, copper, manganese, iron, iodine, selenium), methionine, rosemary extract, cranberry extract, and green tea extract	Fresh fish (cod, common dolphinfish), fresh and dried mutton, cassava starch, black beans, hydrolyzed protein peptides, chicken fat, molasses yeast, dried egg yolk, Taiwan No. 57 dried sweet potato, potato starch, coconut flour, functional cellulose, choline chloride, yeast peptide, sodium tripolyphosphate, taurine, multivitamins (A, B complex, D, E), comprehensive amino acid chelated minerals (zinc, copper, manganese, iron, iodine, selenium), methionine, rosemary extract, cranberry extract, and green tea extract	Dried chicken, fresh chicken, black beans, cassava starch, potato starch, hydrolyzed peptides, chicken fat, dried egg yolk, molasses yeast, fish oil, dried pumpkin, functional cellulose, Taiwan red quinoa, shrimp extract, choline chloride, sodium tripolyphosphate, yeast peptides, taurine, plant fermented peptides, multivitamins (A, B complex, D, E), comprehensive amino acid chelated minerals (zinc, copper, manganese, iron, iodine, selenium), methionine, cranberry extract, and green tea extract

*CA: chicken + fish; CB: fish + mutton; CC: chicken

Table S2. Demographic characteristics of the study group

NO.	Sex	Breed	Neutered	Age (years)	Life stage	Weight (kg) at week 0	CKD stage at week 0	Probiotic pet treats (g/day) [flavor]
PF-1	F*	Mix	Yes	15	Senior	4.05	3	10 [CC]
PF-2	M	Mix	Yes	8	Mature adult	4.65	3	10 [CB]
PF-3	M	Mix	Yes	9	Mature adult	6.30	3	12 [CA]
PF-4	F	Mix	Yes	8	Mature adult	3.09	2	10 [CA]
PF-5	F	Australian domestic longhair	Yes	19	Senior	3.30	3	10 [CA, CC]
PF-6	M	Mix	Yes	19	Senior	5.09	3	10 [CA]
Summary	F: 3 (50%)<br>M: 3 (50%)	Mix: 5 (83.3%)<br>Australian domestic longhair: 1 (16.7%)	Neutered: 6 (100%)<br>Intact: 0 (0%)	13 (8–19)#	Mature adult: 3 (50%)<br>Senior: 3 (50%)	4.41 (3.09–6.30)#	Stage 2: 1 (16.7%)<br>Stage 3: 5 (83.3%)	CA: 3 (50%)<br>CB: 1 (16.7%)<br>CC: 1 (16.7%)<br>Mix: 1 (16.7%)

*F: female; M: male

#The results were displayed as median (range).

Table S3. Detection parameters of gut-derived uremic toxins in LC-MS/MS system

Target	Ion pair Q1 (parent)→Q3 (fragment)	Declustering Potential, DP (volts)	Collision Energy, CE (volts)	Internal standard	Electrospray ionization mode
TMAO	76→58*	70	25	TMAO-d9	Positive
	76→59	70	17		
PCS	187→107*	–41	–30	PCS-d7	Negative
	187→80	–41	–30		
IS	212→80*	–55	–40	IS-d4	Negative
	212→132	–55	–25		
PS	173→93*	–40	–30	PS-^{13}C6	Negative
	173→80	–40	–25		
TMAO-d9	85→66	70	25		Positive
PCS-d7	194→114	–41	–30		Negative
IS-d4	216→80	–55	–40		Negative
PS-^{13}C6	179→99	–40	–30		Negative

Table S4. qPCR primers for targeted microorganisms

Target	Primer	Sequence (5′–3′)	Annealing temperature (°C)	Reference
Bifidobacterium	g-Bifid-F	CTCCTGGAAACGGGTGG	55	1
	g-Bifid-R	GGTGTTCTTCCCGATATCTACA		
Enterobacteriaceae	En-Isu-3F	TGCCGTAACTTCGGGAGAAGGCA	55	2
	En-Isu-3′R	TCAAGGACCAGTGTTCAGTGTC		
Lactobacillus	g-Lact(Rin)-1	AGCAGTAGGGAATCTTCCA	50	3
	g-Lact(Rin)-2	CACCGCTACACATGGAG		
Clostridium perfringens	CIPER-F	AGATGGCATCATCATTCAAC	60	4
	CIPER-R	GCAAGGGATGTCAAGTGT		

Table S5. Harmful residue and pathogenic bacteria analyses of Lm pet treats

Inspection item (ppm)		CA	CB	CC	Criterion
**Heavy metal**	**Arsenic (As)**	0.66	1.38	0.12	< 2 ppm
	**Lead (Pb)**	0.15	0.2	0.14	< 5 ppm
	**Mercury (Hg)**	ND	ND	ND	< 0.4 ppm
	**Cadmium (Cd)**	0.09	0.15	0.04	< 2 ppm
**Aflatoxins**	**Aflatoxins (ppb)**	ND	ND	ND	< 20 ppb
**Pesticides**	**Aldrin and Dieldrin**	ND	ND	ND	< 0.01 ppm
	**Dichloro-diphenyl-trichloroethane (DDT)**	ND	ND	ND	< 0.1 ppm
	**Benzene hexachloride (BHC)**	ND	ND	ND	< 0.01 ppm
	**Endrin**	ND	ND	ND	< 0.01 ppm
	**Heptachlor**	ND	ND	ND	< 0.01 ppm
**Preservatives and antioxidants**	**Ethoxyquin (g/kg food)**	ND	ND	ND	Total amount should < 150 ppm, and Ethoxyquin < 75 ppm
	**Butylated hydroxytoluene (BHT) (g/kg food)**	ND	ND	ND	
	**Butylated hydroxyanisole (BHA) (g/kg food)**	ND	ND	ND	
	**Nitrite (NO2-) (g/kg food)**	ND	ND	ND	< 100 ppm
**Toxic chemical substance**	**Melamine**	ND	ND	ND	< 2.5 ppm
	**Propylene glycol**	< 0.01 %	< 0.01 %	< 0.01 %	Should not detected
**Pathogenic bacteria**	***Salmonella***	–	–	–	–
	***Listeria monocytogenes* (Qualitative)**	–	–	–	–
	**Pathogenic *Escherichia coli***	–	–	–	–
	Clostridium perfringens	–	–	–	–

*CA: chicken + fish; CB: fish + mutton; CC: chicken
*ND: not detected

Table S6. Chemical stability analysis of Lm pet treats

Storage duration (week)	Acid value (mg KOH/g food)			Peroxide value (POV) (mEq/kg food)			Moisture (%)		
	**0**	**4**	**8**	**0**	**4**	**8**	**0**	**4**	**8**
CA	1.43	1.43	1.47	1.3	1.6	1.2	5.1	5.5	6.0
CB	1.38	1.31	1.26	1.9	1.9	1.4	4.7	5.3	5.7
CC	1.92	1.96	1.98	2.5	1.2	1.4	5.3	5.5	6.1

*CA: chicken + fish; CB: fish + mutton; CC: chicken

Table S7. Weight changes in CKD cats throughout the trial

Cat NO./Week	0	4	8
PF-1	4.05	4.08	4.08
PF-2	4.65	4.62	4.64
PF-3	6.30	6.43	6.30
PF-4	3.09	3.18	3.08
PF-5	3.30	3.29	3.26
PF-6	5.09	5.09	4.89
Average	4.413	4.448	4.375

*unit: kg

Table S8. Serum and urinary biochemical parameters of CKD cats during the whole trial

Target	Before Lm intervention	During Lm intervention	After Lm intervention	*P* value	
	week 0	week 4	week 8	week 0 v.s. 4	week 0 v.s. 8
**Hematocrit (%)**	41.04 (34.80–48.90)	39.81 (31.95–49.90)	38.46 (25.75–50.70)	0.230	0.236
**Hemoglobin (g/dL)**	13.47 (11.70–15.90)	13.18 (9.80–16.40)	12.75 (7.60–16.50)	0.401	0.341
**P (mg/dL)**	4.20 (3.40–5.30)	4.53 (3.20–5.90)	4.63 (3.80–6.00)	0.303	0.025
**Ca (mg/dL)**	9.87 (9.20–10.90)	9.52 (8.90–10.10)	9.55 (9.10–10.30)	0.039	0.051
**Ca x P**	41.43 (31.28–52.47)	43.15 (29.37–56.05)	44.08 (39.13–55.20)	0.689	0.115
**UPC**	0.152 (0.07–0.26)	0.125 (0.04–0.29)	0.140 (0.06–0.36)	0.107	0.625
**Specific gravity**	1.013 (1.005–1.022)	1.017 (1.009–1.027)	1.015 (1.009–1.023)	0.128	0.207

*UPC: urine protein/urine creatinine ratio

Data were presented as mean (minimum and maximum). Variables were tested by the ratio paired t test or Wilcoxon sign rank test.

Table S9. Change of CKD stage of tested cats before and after the trial

		week 0			week 8	
**Cat No.**	**creatinine**	**BUN**	**CKD stage**	**creatinine**	**BUN**	**CKD stage**
**PF-1**	2.9	22	3	2.2	31	2
**PF-2**	4.2	38	3	3.8	38	3
**PF-3**	4.3	24	3	3.9	27	3
**PF-4**	2.1	34	2	2.1	21	2
**PF-5**	4	69	3	3.7	74	3
**PF-6**	3.9	40	3	3.6	35	3

*BUN: blood urea nitrogen; CKD: chronic kidney disease

References

1. Matsuki, T.; Watanabe, K.; Fujimoto, J.; Miyamoto, Y.; Takada, T.; Matsumoto, K.; Oyaizu, H.; Tanaka, R. Development of 16S rRNA-gene-targeted group-specific primers for the detection and identification of predominant bacteria in human feces. *Appl. Environ. Microbiol*. **2002**, *68*, 5445–5451. https://doi.org/10.1128/AEM.68.11.5445-5451.2002.
2. Matsuda, K.; Tsuji, H.; Asahara, T.; Kado, Y.; Nomoto, K. Sensitive quantitative detection of commensal bacteria by rRNA-targeted reverse transcription-PCR. *Appl. Environ. Microbiol*. **2007**, *73*, 32–39. https://doi.org/10.1128/AEM.01224-06.
3. Rinttilä, T.; Kassinen, A.; Malinen, E.; Krogius, L.; Palva, A. Development of an extensive set of 16S rDNA-targeted primers for quantification of pathogenic and indigenous bacteria in faecal samples by real-time PCR. *J. Appl. Microbiol.* **2004**, *97*, 1166–1177. https://doi.org/10.1111/j.1365-2672.2004.02409.x.
4. Kikuchi, E.; Miyamoto, Y.; Narushima, S.; Itoh, K. Design of species-specific primers to identify 13 species of *Clostridium* harbored in human intestinal tracts. *Microbiol. Immunol*. **2002**, *46*, 353–358. https://doi.org/10.1111/j.1348-0421.2002.tb02706
